# Associations between socioeconomic status and screen time among children and adolescents in China: A cross-sectional study

**DOI:** 10.1371/journal.pone.0280248

**Published:** 2023-03-23

**Authors:** Youzhi Ke, Sitong Chen, Jintao Hong, Yahan Liang, Yang Liu

**Affiliations:** 1 School of Physical Education, Shanghai University of Sport, Shanghai, China; 2 Institute for Health and Sport, Victoria University, Melbourne, Victoria, Australia; 3 Shanghai Research Institute of Sports Science (Shanghai Anti-Doping Agency), Shanghai, China; 4 Shanghai Research Center for Physical Fitness and Health of Children and Adolescents, Shanghai, China; Khulna University, BANGLADESH

## Abstract

**Background:**

Socioeconomic status (SES) is an important determinant of screen time (ST) in children and adolescents, however, the association between SES and ST is not fully understood in China. This study aimed to investigate the association between SES and ST (operationalized as meeting the ST guidelines; no more than 2 hours per day) in Chinese children and adolescents.

**Methods:**

Cross-sectional data of 2,955 Chinese children and adolescents aged 8 to 17(53.4% girls) were used. SES was measured using indicators of parental education and perceived family wealth. ST was assessed with detailed items from the Health Behaviour School-aged Children survey questionnaires. Descriptive statistics and a Chi-square test were used to report the sample characteristics and analyse ST differences across different sociodemographic groups. A binary logistic regression was then applied to analyse the association of SES indicators with ST in children and adolescents.

**Results:**

Overall, 25.3% of children and adolescents met the ST guidelines. Children and adolescents with higher parental education levels were 1.84 [95% CI 1.31–2.57; father] and 1.42 [95% CI 1.02–1.98; mother] times more likely to meet the ST guidelines than those with lower parental education levels. Associations between SES and ST varied across sex and grade groups. Moreover, the associations of SES with ST on weekdays and weekends were different.

**Conclusions:**

This study demonstrated the association between SES and ST in children and adolescents, highlighting the importance of targeting children and adolescents with low SES levels as an intervention priority. Based on our findings, specific interventions can be tailored to effectively reduce ST. Future studies are encouraged to use longitudinal or interventional designs to further determine the association between SES and ST.

## Introduction

Sedentary behaviour is defined as any waking behaviour characterized by energy of ≤1.5 metabolic equivalents undertaken in a sitting, reclining, or recumbent posture [1]. Screen time (ST), which refers to time spent in watching TV, playing computer games, or playing video games in another way, is an important source of sedentary behaviour, although it does not necessarily reflect total sedentary behaviour time [2]. High levels of ST have become a widespread public health concern [3, 4], as it has been recognized as a health risk factor [5, 6] independent of physical activity (PA) levels [7]. The Canadian 24-Hour Movement Guidelines recommend that children and adolescents limit daily ST no more than 2 hours [8, 9]. Evidence has shown that excessive ST in children and adolescents is associated with various unhealthy behaviours such as irregular sleep [10, 11], eating disorders, poor eating habits [12, 13], as well as physical health outcomes, including obesity, cardiovascular disease, musculoskeletal disease, and higher all-cause mortality [14, 15]. In addition, excessive ST is also strongly associated with a decline in children’s cognitive and social skills [16].

Despite increasing health awareness of ST, high levels of ST remain prevalent in children and adolescents [17, 18]. Guthold et al. compared data from 34 countries, and they found that the percentage of children and adolescents who reported 3 hours or more of ST per day exceeded 30% [19]. In the United States, less than 20% of children and adolescents failed to meet the ST guidelines [20], with ST up to 7.7 hours per day [21]. A similar disappointing situation has been reported in Canada, where children and adolescents spend an average of 8.6 hours per day being sedentary [22]. Children and adolescents also reported an increase in ST in low- and middle-income countries. For example, in China, only 25.5% of children and adolescents meet the ST guidelines [23], with TV viewing time increased from 1 hours per day in 1997 to 1.43 hours per day in 2004 [24], then remaining relatively stable between 2004 and 2011 [25].

ST has become an independent factor negatively affecting the health of children and adolescents [26]. ST habits that develop during childhood and adolescence tend to be maintained in later life, which suggests that ST in early life can predict future ST habits and health outcomes [27, 28]. Given this, ST interventions for children and adolescents should be carried out as early as possible [29]. Researchers studying Chinese population have also found that excessive ST is associated with psychological, emotional, and social problems among children and adolescents [30, 31]. Despite these results, little is known about the association between socioeconomic status (SES) and ST in Chinese children and adolescents.

With the ongoing advancement of technology and rapid changes in lifestyles, ST including watching TV or playing with a mobile phone has become an important part of daily life in young people [32]. Due to the impact of COVID-19, education, both classroom formats and homework have also been changed into online tools, greatly increasing the ST of students [33]. Thus, it is critical to focus efforts on modifiable factors as a means of reducing ST among high-risk groups [34]. ST is affected by multiple factors, among which the influence of SES has received much research attention in recent years [4, 35]. SES, reflects the social class status of individuals, and SES is considered to be an important determinant of health and well-being [36], as it can affect people’s attitudes, experiences, and access to health services [37]. A better understanding of the association between SES and ST can help develop more effective and beneficial strategies to reduce ST. However, the association between SES and ST in children and adolescents has not yet been fully understood, which requires further attention and investigation [27, 38]. A recent systematic analysis found that children and adolescents with lower SES in high-income countries had higher levels of ST compared to those with higher SES, and a similar situation was observed in low- and middle-income countries [38]. Other studies have shown that children and adolescents with lower levels of maternal education levels tend to have more ST than those counterparts with higher levels of maternal education levels [39, 40]. Another study revealed that lower parental education and household income were also associated with higher levels of ST in boys but not in girls [41], while a study from Finland suggested that parental SES was not associated with overall sedentary time. However, it is worth noting that there are some SES differences existing in the proportions of ST and reading time at home [42]. When studying the effects of SES on children’s sedentary behaviour, more attention should be paid to the specific types of sedentary behaviour rather than overall sedentary behaviour. A study from 24 countries in the WHO European region found that low parental education levels and low family perceived wealth were risk factors of watching TV or using electronic devices for at least 2 hours a day, except in Kazakhstan, Kyrgyzstan, Tajikistan, and Turkmenistan [27]. Research on SES and ST in Chinese children is very limited. There is a study from Hong Kong showing that children in lower socioeconomic families were increasingly at risk of sedentary behaviors over the years [43]. Therefore, it is suggested that more studies based on different countries with different social and cultural contexts should be conducted to better understand the association between SES and ST in children and adolescents. However, most previous research focused on people in Western countries, while a few studies were conducted on population in developing countries, particularly in China.

Although results from studies of school-aged children suggested that overall ST is higher during after school periods and on weekends [42], current studies mostly focus on average weekly ST without considering possible differences on weekday and weekend behaviours [44]. It is essential to consider that SES differences in ST occur during weekdays and on weekends in future studies [42]. The aim of this study was to investigate the association between SES and ST in children and adolescents and to evaluate whether the association varied by SES indicators and demographics (e.g., sex).

## Methods

### Study design and sampling procedure

This study conducted a cross-sectional survey of schools in China’s provinces of Jiangsu, Anhui, Zhejiang, and Shanghai from September to December 2019. A multistage sampling approach was used to recruit study respondents from students in primary, junior middle, and high schools. However, because students below third grade were not considered to be able to read the questionnaire, only healthy students of 3^rd^ to 12^th^ grades were included in this study. Exclusion criteria were children and adolescents who were nonverbal or ill and whose first language was not Chinese. In the first step, a total of 34 primary, junior middle, and high schools from Jiangsu, Anhui, Zhejiang, and Shanghai were selected using a convenient sample approach. In the second stage, a random cluster sampling was used to select classes in the target grades within these schools. This study was approved by the Institutional Review Board (IRB) of Shanghai University of Sport (SUS), and because none of the survey items was concerned with any personal or ethical issues, the IRB determined that verbal assent from participation was sufficient. Consequently, the necessity for written consent was waived.

### Participants

Participants were 3,368 students from the selected primary schools (3^rd^ to 6^th^ grades, aged 8 to 11 years old, n = 527), junior middle schools (7^th^ to 9^th^ grades, 12 to 14 years old, n = 1,809), and high schools (10^th^ to 12^th^ grades, 15 to 17 years old, n = 619) schools, with participants ranging in age from 8 to17 years old. The self-reported questionnaire was completed by 2,955 students (response rate = 87.7%).

### Procedures

Teachers and principals of the participating schools allowed the research staff to conduct the study. All children and adolescents involved in the study, together with their parents or guardians, were informed that participation was entirely voluntary, that verbal informed consent was obtained from all parents or guardians, and affirmative consent was obtained from all children and adolescents prior to data collection. Trained research assistants pre-arranged the survey in accordance with a standardized administration protocol during regular school hours, and the survey was thus completed on paper in the classroom setting. Students were instructed on how to complete the survey and given ample time to fill in the questionnaire. Data from the survey was then collected and analysed anonymously.

#### Measurements

*Sociodemographic*. Apart from body height and weight, all measures used in this study were based on self-report from the survey questionnaire. Children and adolescents were asked to report their demographic information, including sex (1 = boy, 2 = girl), age, and grade (3rd to 12^th^ grades), and ethnicity (Han or other). Among children under 10 years, questionnaires were completed with the assistance of trained research assistants. Further details on each measure used in this study were provided below.

*Screen time*. ST was measured by reliable and valid items derived from the Health Behaviour in School-aged Children instrument [45]. TV time was assessed by using the question "How many hours do you usually spend watching television in your free time?", with separations for weekdays and weekends (reliability coefficients of 0.74 and 0.72, respectively). Computer time was assessed by using the question "How many hours do you usually spend using a computer or game console (such as PS, Wii, Xbox, etc.) to surf the Internet or play games in your free time?", again for weekdays and weekends separately (reliability coefficients of 0.54 and 0.69, respectively). Smartphone time was assessed by the question "How many hours do you usually spend using electronic products such as tablets or smartphones to surf the Internet or play games in your free time?", again for weekdays and weekends separately (reliability coefficients of 0.33 and 0.50, respectively). The available responses to each question were "none", "about 0.5 h", "1 h", "2 h", or "3 h or more". Total ST was then calculated by summing up all answers from questions (TV, computer, smartphone time, etc). According to the Canadian 24-Hour Movement Guidelines, meeting the ST guideline requires a total daily ST ≤ 2h per day [8].

*Socioeconomic status*. Individual SES measures were adopted based on both parental education and a measure of perceived family wealth assessments [46]. Parental education level was determined based on reported data, categorizing parents’ educational experience into seven groups: 1) Below elementary school; 2) Elementary school; 3) Junior middle school; 4) High school or occupational school; 5) College; 6) Undergraduate; and 7) Postgraduate and above. Parental education level were further divided into three categories for analysis: a low education level (below the elementary school, elementary school, and junior middle school), a medium education level (high school or occupational school and college), and a high level of education (undergraduate or postgraduate and above).

The perceived family wealth was assessed by the study participants’ perceptions towards their family’s current SES. This variable was developed from the question "How well off do you think your family is?" with the available response of "very well off", "quite well off", "average", "not very well off", and "not at all well off". In the analysis, perceived family wealth was further divided into three categories: a low economic level (not very well off and not at all well off), a medium economic level (average), and a high economic level (very well off and quite well off).

### Statistical analyses

All the statistical analysis was performed using SPSS 24.0 version. All missing cases and abnormal values were removed. Considering the Chinese educational system, grade groups are divided into primary, junior middle, and high schools. Descriptive statistics were used to report the sample characteristics, with continuous variables expressed as mean ± standard deviation, and categorical variables expressed as numbers (n) or with percentages (%). Between-group differences in categorical demographic variables were tested by using a chi-square test. Binary logistic regression was used to analyse the association between SES indicators and ST, adjusted for sociodemographic factors. All logistic regression analysis results were presented as odds ratios (OR) with a 95% confidence interval (CI). All *p ≤* 0.05 were considered to be statistically significant.

## Results

The descriptive characteristics of the analytical sample in this study are shown in Table 1. A total of 2,955 children and adolescents (53.4% girls) were included in the final analysis, with an average age of 13.36 ± 2.46 years (13.08 ± 2.43 of boys and 13.01 ± 2.47 of girls, *p* < 0.001). Participants from primary school, junior middle school, and high school accounted for 17.9%, 61.2%, and 20.9%, respectively. There was a statistically significant sex difference between grade groups (*p* < 0.001). The majority of participants were Han Chinese (96.9%), and no significant difference was found between ethnic groups (*p > 0*.*05)*. About half of the participants reported that their fathers and mothers had low levels of education (41.0% and 47.7%, *p* > 0.05), respectively, and 57.5% of the participants had medium perceived family wealth (55.2% for boys, and 59.5% for girls, *p* < 0.05).

**Table 1:** The characteristics of the samples.

			Overall (2955)	Boys (1378)	Girls (1577)	*P*
**Age (years), M±SD**			13.36±2.46	13.08±2.43	13.01±2.47	<0.001
**Grade groups, n (%)**						<0.001
	Primary school		527(17.9)	269(19.5)	258(16.4)	
	Junior middle school		1809(61.2)	934(67.8)	875(55.5)	
	High school		619(20.9)	175(12.7)	444(28.2)	
**Ethnicity, n (%)**						0.071
	Han		2862(96.9)	1399(97.2)	1523(96.6)	
	Others		93(3.2)	39(2.9)	54(3.4)	
**SES, n (%)**						
	*Paternal education level*					0.345
		Low	1211(41.0)	546(39.6)	665(42.2)	
		Medium	1021(34.6)	483(35.1)	538(341)	
		High	723(24.4)	349(25.3)	374(23.7)	
	*Maternal education level*					0.113
		Low	1409(47.7)	629(45.6)	780(49.5)	
		Medium	899(30.4)	433(31.4)	466(29.5)	
		High	647(21.9)	316(22.9)	331(21.0)	
	*Perceived family wealth*					0.002
		Low	333(11.3)	143(10.4)	190(12.0)	
		Medium	1699(57.5)	760(55.2)	939(59.5)	
		High	923(31.2)	475(34.5)	448(28.4)	

Notes: M, Means; SD, standard deviation; SES: socioeconomic status.

Primary school: 8–11 years

Junior middle school: 12–14 years

High school: 15–17 years

Table 2 shows the prevalence of ST by sex and grade group. Overall, approximately a quarter (25.3%) of children and adolescents met ST guidelines, with 81.5% and 37.9% meeting ST guidelines on weekdays and weekends, respectively. The percentage of boys meeting ST guidelines was higher than that of girls both in total and on weekends (25.6% vs 25.1% and 39.0% vs 36.9%, respectively). On weekdays, the percentage of girls meeting ST guidelines was higher than that of boys (84.0% vs 78.7%, *p* < 0.001). Percentages meeting the ST guidelines across the three grade groups significantly differed (primary school, 34.9%; junior middle school, 28.7%; high school:7.3%; *p* < 0.001).

**Table 2:** Prevalence of meeting screen time guidelines.

Category		Total ST ^b^		Weekday ST ^a^^,^^b^		Weekend ST ^b^	
		*Not Meet (%)*	*Meet (%)*	*Not Meet (%)*	*Meet (%)*	*Not Meet (%)*	*Meet (%)*
**Total**		74.7	25.3	18.5	81.5	62.1	37.9
**Sex**							
	Boys	74.4	25.6	21.3	78.7	61.0	39.0
	Girls	74.9	25.1	16.0	84.0	63.1	36.9
**Grade**							
	Primary school	65.1	34.9	26.6	73.4	39.5	60.5
	Junior middle school	71.3	28.7	14.5	85.5	62.0	38.0
	High school	92.7	7.3	23.3	76.7	81.9	18.1

ST: screen time

^a^denotes significant sex difference at *p* < 0.001

^b^denotes significant grade group difference at *p* < 0.001

Primary school: 8–11 years

Junior middle school: 12–14 years

High school: 15–17 years

**Fig 1:**
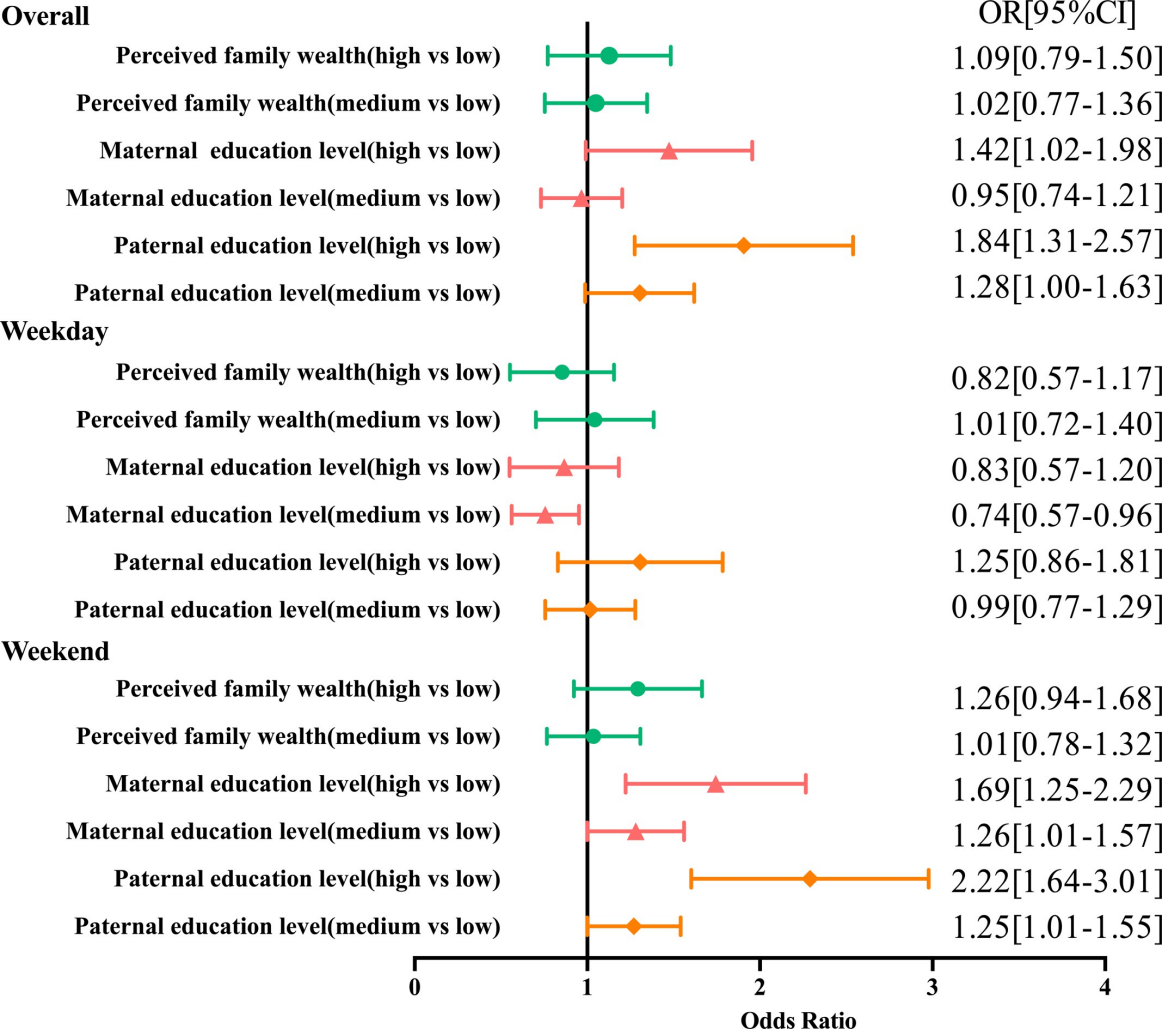
Regression analysis of socioeconomic status and screen time.

Associations between SES and the prevalence of meeting the ST guidelines are shown in Fig 1. Participants with medium and high paternal education levels were 1.28 [95% CI 1.00–1.63] and 1.84 [95% CI 1.31–2.57] times more likely to spend less than 2 hours a day on watching TV or using electronic devices than those with low paternal education levels, respectively. Participants with high maternal education levels were 1.42 [95% CI 1.02–1.98] times more likely to meet ST guidelines than participants with low maternal education levels. Similarly, participants whose fathers had medium and high education levels were 1.25 [95% CI 1.01–1.55] and 2.22 [95% CI 1.64–3.01] times more likely to meet ST guidelines than participants whose fathers had low education levels on weekends, respectively. Participants whose mothers had medium and high education levels were 1.26 [95% CI 1.01–1.57] and 1.69 [95% CI 1.25–2.29] times more likely to meet ST guidelines than participants whose mothers had low education levels on weekends, respectively.

**Fig 2:**
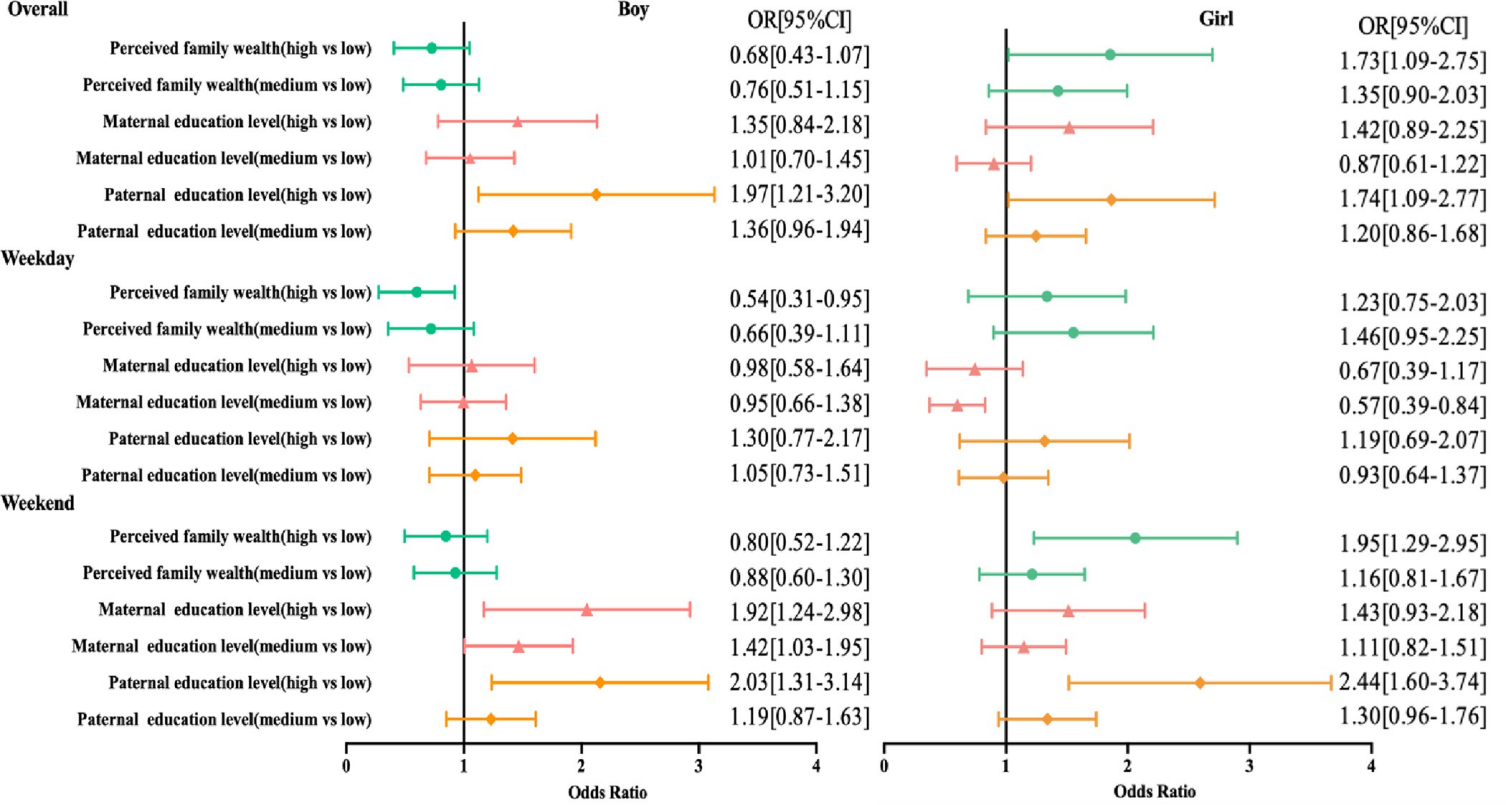
Regression analysis of sex differences in socioeconomic status and screen time.

The summary results of OR for participants meeting ST guidelines by sex are shown in Fig 2. Both boys and girls with high paternal education levels were 1.97 [95% CI 1.21–3.20] and 1.74 [95% CI 1.09–2.77] times more likely to meet ST guidelines than participants with low paternal education levels, respectively. Girls with high perceived family wealth were 1.73 [95% CI 1.09–2.75] and 1.95 [95% CI 1.29–2.95] times more likely to spend no more than 2 hours per day on ST overall and on weekends, respectively. Boys with high perceived family wealth were more likely to spend more than 2 hours on ST per day on weekdays (OR 0.54 (95% CI 0.31–0.95)). Girls with medium maternal education levels were more likely to spend more than 2 hours per day on ST than girls with low maternal education levels on weekdays (OR 0.57 (95% CI 0.39–0.84)). Both boys and girls with high paternal education levels were 2.03 [95% CI 1.31–3.14] and 2.44 [95% CI 1.60–3.74] times more likely to meet the ST guidelines than participants with low paternal education levels on weekends, respectively. Boys with medium and high maternal education levels were 1.42 [95% CI 1.03–1.95] and 1.92 [95% CI 1.24–2.98] times more likely to meet ST guidelines than boys with low maternal education levels, respectively.

**Fig 3:**
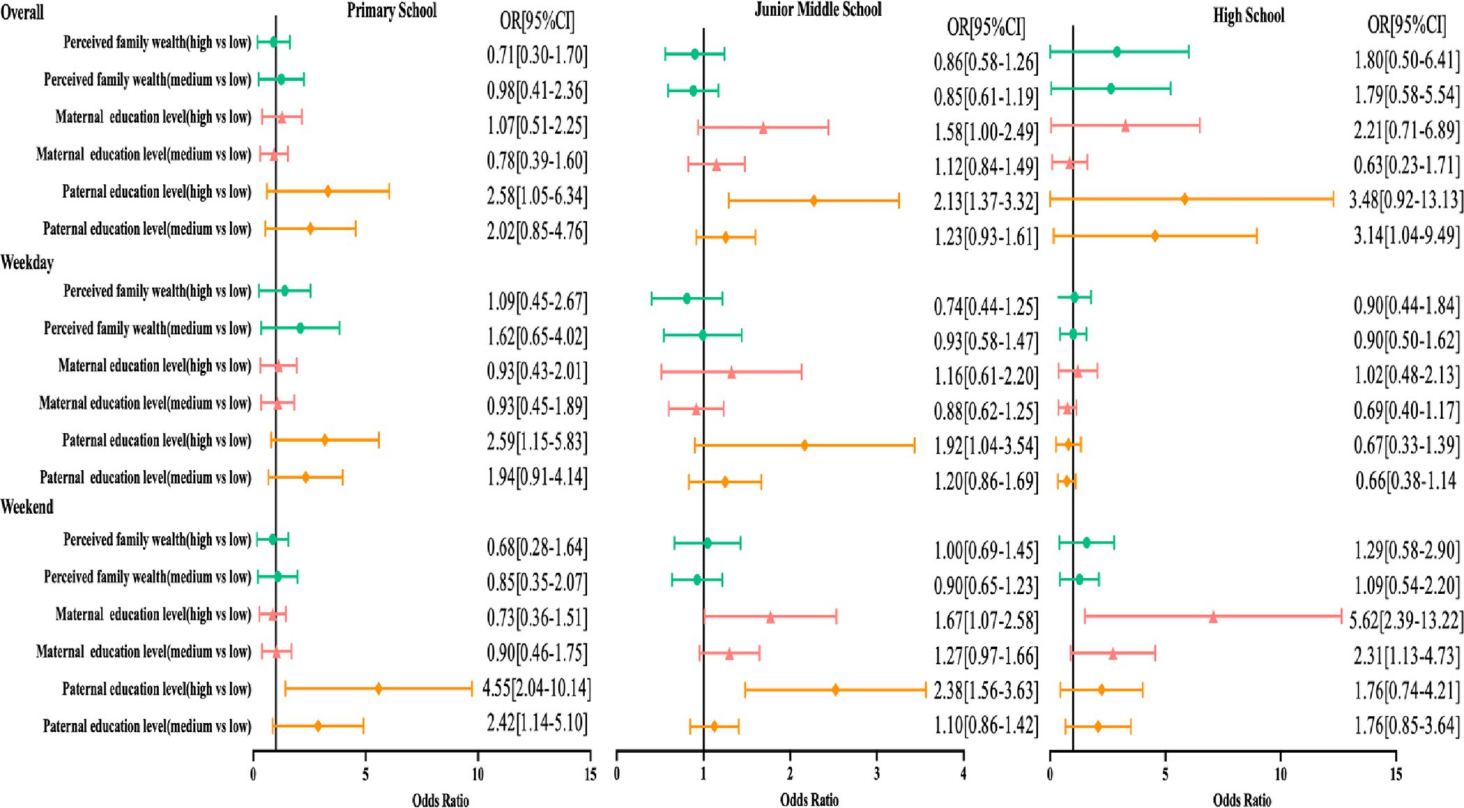
Regression analysis of grade differences in socioeconomic status and screen time.

The summary results of OR for participants meeting the ST guidelines by grade group are shown in Fig 3. Participants from primary school students and junior middle school students with high paternal education levels were more likely to spend no more than 2 hours per day on ST than participants with low paternal education levels on weekdays and weekends. Participants from junior middle school students and high school students with high maternal education levels were more likely to spend no more than 2 hours per day on ST than participants with low maternal education levels on weekends (OR = 1.67,95%CI:1.07–2.58; OR = 5.62,95%CI:2.39–13.22, respectively). Participants from high school students with high paternal education levels were 3.14 [95% CI 1.04–9.49] times more likely to meet ST guidelines than participants with low paternal education levels.

## Discussion

This study investigated the association between SES and ST in Chinese children and adolescents. The main findings of this study are that children and adolescents with low parental education levels spent more ST on weekends than their counterparts with highly educated parents, with the difference being particularly notable for parental education levels. Various SES indicators also show different associations with ST across sex and grade groups of children and adolescents.

The underlying mechanism explaining the association between parental education level and ST may be that parents with higher education levels are more aware of the health impacts of excessive ST and pay more attention to their children’s academic development, thus limiting children’s and adolescent’s time spent in front of screen-based devices and encouraging to participate in physical activity [42]. However, less educated parents probably ignore the health effects of ST, and may thus tend to be less likely to limit their children’s ST [47]. Some research has suggested that parents have important modelling roles to their children, which can directly affect their children’s behaviours, such as preference for ST or participation in physical activity [33, 48]. Parental role models, attitudes, and awareness can thus have an impact on ST in children and adolescents, and this should be considered when aiming to reduce SES differences across ST in children and adolescents.

In addition, parents with higher education levels are more likely to provide more financial supports to help their children participate in physical activity, thereby potentially reducing ST [23, 49], while parents with lower education levels may not be able to provide such supports, making it challenging for such parents to limit children’s ST [42, 50]. Moreover, children with less educated parents may have more time unsupervised at home because of parents’ prolonged working hours, and this could increase their children’s longer exposure to ST [44]. It should be noted that, however, this study showed that paternal education levels appear to have a greater impact on ST in children and adolescents than maternal education levels, suggesting that priority should be given to groups with lower paternal education levels in future interventions.

The increasing use of electronic screen-based devices is another contributory factor to explain the findings. Previous studies have shown that children and adolescents with low SES are more likely to have televisions and video game systems in their bedrooms [34, 35], a practice that is associated with higher ST levels [51, 52]. However, a recent meta-analysis found that the associations between SES and ST in children and adolescents mainly depend on the country context, with SES being inversely related to ST in high-income countries and positively related to it in low- and middle-income countries [38]. This means that different intervention approaches should be formulated according to the specific social and cultural contexts. Based on this, there may be a need to counter-market electronic products such as gaming devices to low socio-economic households, especially for younger children.

This study showed that boys and girls with high levels of paternal education levels have lower ST throughout the whole week. Potential reasons for these findings include the fact that fathers with higher levels of education and social status may be more aware of the health consequences of excessive ST and thus have stricter rules on children’s ST behaviours [42]. Previous studies have suggested that fathers with lower levels of SES have fewer regulations around their children’s television access, as well as watching television more often with their children [34, 53]. Based on this, children may engage in more ST. Future interventions to reduce SES differences in children’s ST may thus need to focus on parental regulations and limits on children’s ST [42, 43].

An interesting finding in this work was that the higher levels of maternal education was associated with less ST among boys while more ST among girls. This result was inconsistent with previous findings [12, 54]. Cultural and lifestyle differences between developed and developing countries may help explain these differences. Girls whose mothers have higher education levels may be encouraged to spend more time on educational ST, such as drawing with electronics, than girls with less educated mothers [55]. Moreover, girls are seen as more vulnerable to exposure to screen-based devices than boys, and mothers are more concerned about girls’ safety, while boys are more likely to be encouraged in sports activities on weekends [56].

Results from previous studies on Chinese adolescent showed that any form of parental support, including verbal encouragement and additional parental presence, linked less time spent on ST [57]. Raising parents’ awareness of ST limit should be a priority to reduce sedentary behaviours in children with lower levels of SES. In addition, this study showed that girls with lower levels of perceived family wealth had more ST. This perhaps because parents with low levels of SES are more concerned about the safety of their neighbourhoods [58, 59], as well as lacking time to supervise their children in neighbourhoods [60] and have fewer opportunities and resources to encourage their children to engage in physical activity [61], leading to indoor screen-based activities [62]. Overall, the current findings suggest that boys with lower levels of parental education are a good target group for ST reduction, while for girls, more attention needs to be paid to the impact of maternal education. This highlights the importance of taking parental SES status into account when implementing interventions for ST reductions in children and adolescents [41], although such information may help to target and design more effective family-based interventions to reduce socioeconomic outcomes for both boys and girls [42].

Different school stages represent different grade groups of study participants, and these different grade groups showed several differences in ST. This research showed that the higher levels of parental education, less ST occurs in primary and junior middle school students, mainly on weekends. For primary school students, only paternal education level showed a positive association with ST. However, in a previous Finnish study, maternal education had no impacts, while highly educated fathers were associated with less ST in children. Yet less educated fathers were not associated with ST in their children [63]. In contrast to the work of Maatta et al. [42], the current study suggested that paternal education level potentially had an impact on ST in children and adolescents of different ages, while maternal education level had no significant impact on ST in children and adolescents of younger ages. Thus, that fathers could have a more profound impact on ST in children and adolescents, while mothers are important to affect ST in older children and adolescents. In China, children and adolescents from primary school may find their father’s role to be more important to their learning and health, their mothers are likely to have a greater direct impact on their lives due to different cultural roles. Fathers with higher education levels are more likely to be aware of the harms of ST and thus more likely to encourage their children to participate in physical activity [64]. As children and adolescents from junior middle school develop self-awareness, they are, however, likely to participate in more activities autonomously. Many schools are thus now promoting post-secondary education and training after high school in China, reducing the possibility of ST on weekdays for students. These supportive high schools have a certain relationship with parental education. However, families with high parental education levels are more inclined to make efforts to make their children into better schools [54]. Additionally, participants from high school students with higher SES are more likely to participate in many weekend clubs on weekends, while participants with lower SES have more ST [65]. Affording additional clubs and training is a major challenge for families with low SES, highlighting the need to focus any intervention on families with low-educated fathers, based on their influence on children’s ST on weekends.

## Strengths and limitations

This study includes some strengths, such as a relatively equal distribution of samples across different demographic groups (e.g., sex, grade), a large sample size that can increase the generalizability of findings. However, there are some limitations of this study. As data were collected by a self-reported questionnaire, this may be affected by respondents’ recall bias. The cross-sectional design also cannot draw a cause-and-effect association between SES and ST. Future studies should apply improved methodological approaches to further determine the association between SES and ST.

## Conclusion

The most consistent finding from this study is that children and adolescents with lower SES are more likely to have higher levels of ST in China. It would therefore be worthwhile to develop strategies to reduce ST that focus on these children and adolescents. The findings also illustrate the multidimensionality of the relationship between ST and SES in children and adolescents, including multiple ST measurements and multiple SES indicators. Moreover, taking into account the various contexts emerging over the course of a week, including weekdays and weekends, our findings would deepen understanding of the association between SES and ST in children and adolescents.

This study suggest that work to limit ST is urgently needed among children and adolescents with low SES in China, as this may improve their future health outcomes. Activities on weekends for children and adolescents with low parental education levels should be targeted as a priority.

## Supporting information

S1 Data (XLSX)

## References

[1] Tremblay MS, Aubert S, Barnes JD, Saunders TJ, Arto PJ. Sedentary Behavior Research Network (SBRN)–Terminology Consensus Project process and outcome. International Journal of Behavioral Nutrition and Physical Activity. 2017;14(75):2–17. 28599680 10.1186/s12966-017-0525-8 PMC5466781

[2] Maïté V, Van LW, Lea M, Mine Y, Mai C, Yannis M, et al. Self-reported TV and computer time do not represent accelerometer-derived total sedentary time in 10 to 12-year-olds. European Journal of Public Health. 2013;(1):30–2. doi: 10.1093/eurpub/cks047 22544913

[3] Hu Y, Kirk JG, Wu H. Screen time relationship of Chinese parents and their children. Children and Youth Services Review. 2018;94:S0190740918303621.

[4] Dong XS, Ding LJ, Zhang R, Ding M, Wang BZ, Yi XR. Physical Activity, Screen-Based Sedentary Behavior and Physical Fitness in Chinese Adolescents: A Cross-Sectional Study. Frontiers in Pediatrics. 2021;9. doi: 10.3389/fped.2021.722079 34676185 PMC8524360

[5] Tremblay MS, Colley RC, Saunders TJ, Healy GN, Owen N. Physiological and health implications of a sedentary lifestyle. Applied physiology, nutrition, and metabolism. 2010;35(6):725–40. doi: 10.1139/H10-079 21164543

[6] Yang-Huang J, van Grieken A, Wang L, Jansen W, Raat H. Clustering of Sedentary Behaviours, Physical Activity, and Energy-Dense Food Intake in Six-Year-Old Children: Associations with Family Socioeconomic Status. Nutrients. 2020;12(6):1–13. doi: 10.3390/nu12061722 32526862 PMC7352876

[7] Salmon J, Tremblay MS, Marshall SJ, Hume C. Health Risks, Correlates, and Interventions to Reduce Sedentary Behavior in Young People. American Journal of Preventive Medicine. 2011;41(2):197–206. doi: 10.1016/j.amepre.2011.05.001 21767728

[8] Tremblay MS, Carson V, Chaput JP, Gorber SC, Zehr L. Canadian 24-Hour movement guidelines for children and youth: An integration of physical activity, sedentary behaviour, and sleep. Applied Physiology, Nutrition, and Metabolism. 2016;41. 10.1139/apnm-2016-0203 27306430

[9] World Health Organization. WHO guidelines on physical activity and sedentary behaviour. Geneva. 2020. 33369898

[10] Wu X, Tao S, Rutayisire E, Chen Y, Huang K, Tao F. The relationship between screen time, nighttime sleep duration, and behavioural problems in preschool children in China. European Child & Adolescent Psychiatry. 2017;26(5):541–8. doi: 10.1007/s00787-016-0912-8 27822641

[11] Matricciani LA, Olds TS, Blunden S, Rigney G, Williams MT. Never Enough Sleep: A Brief History of Sleep Recommendations for Children. Pediatrics. 2012;129(3):548–56. doi: 10.1542/peds.2011-2039 22331340

[12] Cardenas-Fuentes G, Homs C, Ramirez-Contreras C, Juton C, Casas-Esteve R, Grau M, et al. Prospective Association of Maternal Educational Level with Child’s Physical Activity, Screen Time, and Diet Quality. Nutrients. 2022;14(1):1–10. 10.3390/nu14010160 PMC8747409 35011035

[13] Barr-Anderson DJ, Van Den Berg P, Neumark-Sztainer D, M S. Characteristics Associated With Older Adolescents Who Have a Television in Their Bedrooms. Pediatrics. 2008. doi: 10.1542/peds.2007-1546 18381536

[14] Stewart R, Benatar J, Maddison R. Living longer by sitting less and moving more. Current Opinion in Cardiology. 2015;30(5):551. doi: 10.1097/HCO.0000000000000207 26204494

[15] Tremblay MS, LeBlanc AG, Kho ME, Saunders TJ, Larouche R, Colley RC, et al. Systematic review of sedentary behaviour and health indicators in school-aged children and youth. International Journal of Behavioral Nutrition & Physical Activity. 2011;8(1): 1–22. doi: 10.1186/1479-5868-8-98 21936895 PMC3186735

[16] Tomopoulos S, Dreyer BP, Berkule S, Fierman AH, Mendelsohn AL. Infant Media Exposure and Toddler Development. JAMA Pediatrics. 2010;164(12):1105–11. doi: 10.1001/archpediatrics.2010.235 21135338 PMC3095486

[17] Xiao Q, Keadle SK, Berrigan D, Matthews CE. A prospective investigation of neighborhood socioeconomic deprivation and physical activity and sedentary behavior in older adults. Preventive Medicine. 2018;111:14–20. doi: 10.1016/j.ypmed.2018.02.011 29454077 PMC6485255

[18] Zhu Z, Tang Y, Zhuang J, Liu Y, Wu X, Cai Y, et al. Physical activity, screen viewing time, and overweight/obesity among Chinese children and adolescents: an update from the 2017 physical activity and fitness in China—the youth study. BMC Public Health. 2019; 19(1): 1–8. 30767780 10.1186/s12889-019-6515-9 PMC6376726

[19] Guthold R, Cowan MJ, Autenrieth CS, Kann L, Riley LM. Physical activity and sedentary behavior among schoolchildren: a 34-country comparison. Journal of Pediatrics. 2010;157(1):43–9.e1. doi: 10.1016/j.jpeds.2010.01.019 20304415

[20] Herrick KA, Fakhouri TH, Carlson SA, Fulton JE. TV watching and computer use in U.S. youth aged 12–15, 2012. Nchs Data Brief. 2014;157(157):1–8. 25007319

[21] Piercy KL, Troiano RP, Ballard RM, Carlson SA, Fulton JE, Galuska DA, et al. The Physical Activity Guidelines for Americans. Jama-Journal of the American Medical Association. 2018;320(19):2020–8. 10.1001/jama.2018.14854 PMC9582631 30418471

[22] Colley RC, Garriguet D. Physical activity and sedentary behavior during the early years in Canada: a cross-sectional study. The International Journal of Behavioral Nutrition and Physical Activity. 2013;10 (1): 1–9. doi: 10.1186/1479-5868-10-54 23642258 PMC3655822

[23] Chen ST, Liu Y, Hong JT, Tang Y, Cao ZB, Zhuang J, et al. Co-existence of physical activity and sedentary behavior among children and adolescents in Shanghai, China: Do gender and age matter? BMC Public Health. 2018;18(1):1287–1296. doi: 10.1186/s12889-018-6167-1 30466431 PMC6251113

[24] Zhang J, Seo DC, Kolbe L, Middlestadt S, Zhao W. Associated Trends in Sedentary Behavior and BMI Among Chinese School Children and Adolescents in Seven Diverse Chinese Provinces. International Journal of Behavioral Medicine. 2012;19(3):342–50. doi: 10.1007/s12529-011-9177-2 21748473

[25] Dearth-Wesley T, Howard AG, Wang H, Zhang B, Popkin BM. Trends in domain-specific physical activity and sedentary behaviors among Chinese school children, 2004–2011. International Journal of Behavioral Nutrition and Physical Activity. 2017;14(1): 1–9. 29058623 10.1186/s12966-017-0598-4 PMC5651590

[26] Saunders TJ, Gray CE, Poitras VJ, Chaput J-P, Janssen I, Katzmarzyk PT, et al. Combinations of physical activity, sedentary behaviour and sleep: relationships with health indicators in school-aged children and youth. Applied Physiology Nutrition and Metabolism. 2016;41(6):S283–S93. doi: 10.1139/apnm-2015-0626 27306434

[27] Music Milanovic S, Buoncristiano M, Krizan H, Rathmes G, Williams J, Hyska J, et al. Socioeconomic disparities in physical activity, sedentary behavior and sleep patterns among 6-to 9-year-old children from 24 countries in the WHO European region. Obesity Reviews. 2021;22:e13209. doi: 10.1111/obr.13209 34235843

[28] Mcveigh JA, Zhu K, Mountain J, Pennell CE, Lye SJ, Walsh JP, et al. Longitudinal Trajectories of Television Watching Across Childhood and Adolescence Predict Bone Mass at Age 20 Years in the Raine Study. Journal of Bone & Mineral Research. 2016;31 (11): 2032–2040. doi: 10.1002/jbmr.2890 27378122

[29] Pearson N, Griffiths P, van Sluijs E, Atkin AJ, Khunti K, Sherar LB. Associations between socioeconomic position and young people’s physical activity and sedentary behaviour in the UK: a scoping review. BMJ open. 2022;12(5):e051736. doi: 10.1136/bmjopen-2021-051736 35501089 PMC9062792

[30] Li S, Lester A, Fan J, Chen W, Jin X, Yan C, et al. Sleep, School Performance, and a School-Based Intervention among School-Aged Children: A Sleep Series Study in China. Plos One. 2013;8(7):e67928. doi: 10.1371/journal.pone.0067928 23874468 PMC3707878

[31] Zhang YB, Harwood J. Television Viewing and Perceptions of Traditional Chinese Values Among Chinese College Students. Journal of Broadcasting & Electronic Media. 2002;46(2):245–64.

[32] Liu M, Wu L, Yao S. Dose–response association of screen time-based sedentary behaviour in children and adolescents and depression: a meta-analysis of observational studies. British Journal of Sports Medicine. 2016;50(20):1252–8. doi: 10.1136/bjsports-2015-095084 26552416 PMC4977203

[33] Stienwandt S, Cameron EE, Soderstrom M, Casar M, Le C, Roos LE. Family Factors Associated with Hands-On Play and Screen Time During the COVID-19 Pandemic. Child & Youth Care Forum. 2022; 1–25. doi: 10.1007/s10566-021-09668-4 35013660 PMC8731198

[34] Tandon PS, Zhou C, Sallis JF, Cain KL, Frank LD, Saelens BE. Home environment relationships with children’s physical activity, sedentary time, and screen time by socioeconomic status. International Journal of Behavioral Nutrition and Physical Activity. 2012; 9(1): 1–9. doi: 10.1186/1479-5868-9-88 22835155 PMC3413573

[35] Rodrigues D, Gama A, Machado-Rodrigues AM, Nogueira H, Rosado-Marques V, Silva MRG, et al. Home vs. bedroom media devices: socioeconomic disparities and association with childhood screen- and sleep-time. Sleep Medicine. 2021;83:230–4. doi: 10.1016/j.sleep.2021.04.012 34049041

[36] Shavers VL. Measurement of socioeconomic status in health disparities research. Journal of the national medical association. 2007;99(9):1013–20. 17913111 PMC2575866

[37] Marmot M. Inclusion health: addressing the causes of the causes. Lancet (London, England). 2018;391(10117):186–8. doi: 10.1016/S0140-6736(17)32848-9 29137870

[38] Mielke GI, Brown WJ, Nunes BP, Silva I, Hallal PC. Socioeconomic Correlates of Sedentary Behavior in Adolescents: Systematic Review and Meta-Analysis. Sports Medicine. 2017;47(1):61–75. doi: 10.1007/s40279-016-0555-4 27260683 PMC5215067

[39] Wrnberg J, Pérez-Farinós N, Benavente-Marín J, Gómez S, Barón-López F. Screen Time and Parents’ Education Level Are Associated with Poor Adherence to the Mediterranean Diet in Spanish Children and Adolescents: The PASOS Study. Journal of clinical medicine. 2021;10(4):795. doi: 10.3390/jcm10040795 33669366 PMC7920265

[40] Pons M, Bennasar-Veny M, Yaez AM. Maternal Education Level and Excessive Recreational Screen Time in Children: A Mediation Analysis. International Journal of Environmental Research and Public Health. 2020;17(23):8930. doi: 10.3390/ijerph17238930 33271768 PMC7730269

[41] Lampinen E-K, Eloranta A-M, Haapala EA, Lindi V, Väistö J, Lintu N, et al. Physical activity, sedentary behaviour, and socioeconomic status among Finnish girls and boys aged 6–8 years. European journal of sport science. 2017;17(4):462–72. doi: 10.1080/17461391.2017.1294619 28276910

[42] Maatta S, Konttinen H, Haukkala A, Erkkola M, Roos E. Preschool children’s context-specific sedentary behaviours and parental socioeconomic status in Finland: a cross-sectional study. Bmj Open. 2017;7(11): e016690. doi: 10.1136/bmjopen-2017-016690 29101133 PMC5695314

[43] Gong WJ, Fong DYT, Wang MP, Lam TH, Chung TWH, Ho SY. Increasing socioeconomic disparities in sedentary behaviors in Chinese children. Bmc Public Health. 2019;19:(1): 1–10. 31196044 10.1186/s12889-019-7092-7 PMC6567653

[44] Lehto E, Lehto R, Ray C, Pajulahti R, Sajaniemi N, Erkkola M, et al. Are associations between home environment and preschool children’s sedentary time influenced by parental educational level in a cross-sectional survey? International Journal for Equity in Health. 2021; 20(1): 1–11. 33422074 10.1186/s12939-020-01333-x PMC7796557

[45] Liu Y, Mei, Wang, Jorma, Tynjälä, Yan, et al. Test-retest reliability of selected items of Health Behaviour in School-aged Children (HBSC) survey questionnaire in Beijing, China. BMC Medical Research Methodology. 2010; 10(1): 1–9. doi: 10.1186/1471-2288-10-73 20696078 PMC2927607

[46] Liu Y, Mei, Wang, Jari, Villberg, Torbjørn, et al. Reliability and Validity of Family Affluence Scale (FAS II) among Adolescents in Beijing, China. Child Indicators Research. 2012; 5(2): 235–251.

[47] Pate RR, Mitchell JA, Byun W, Dowda M. Sedentary behaviour in youth. Br J Sports Med. 2011;45(11):906–13. doi: 10.1136/bjsports-2011-090192 21836174

[48] Hong J, Chen S, Tang Y, Cao ZB, Liu Y. Associations between Various Kinds of Parental Support and Physical Activity among Children and Adolescents in Shanghai, China: Gender and Age Differences. BMC Public Health. 2020; 20(1): 1–9. 32711483 10.1186/s12889-020-09254-8 PMC7382138

[49] Moradi G, Mostafavi F, Azadi N, Esmaeilnasab N, Nouri B. Evaluation of screen time activities and their relationship with physical activity, overweight and socioeconomic status in children 10–12 years of age in Sanandaj, Iran: A cross-sectional study in 2015. Medical Journal of the Islamic Republic of Iran. 2016;30(1):448. 28210613 PMC5307628

[50] Bentley GF, Turner KM, Jago R. Mothers’ views of their preschool child’s screen-viewing behaviour: a qualitative study. BMC Public Health. 2016;16(1):1–11. doi: 10.1186/s12889-016-3440-z 27492488 PMC4973523

[51] Dumuid D, Olds TS, Lewis LK, Maher C. Does home equipment contribute to socioeconomic gradients in Australian children’s physical activity, sedentary time and screen time? BMC Public Health. 2016;16(1): 1–8. doi: 10.1186/s12889-016-3419-9 27496020 PMC4975892

[52] Gilbert-Diamond D, Li Z, Adachi-Mejia AM, Mcclure AC, Sargent JD. Association of a television in the bedroom with increased adiposity gain in a nationally representative sample of children and adolescents. Jama Pediatrics. 2014;168(5):427–434. doi: 10.1001/jamapediatrics.2013.3921 24589630 PMC4141563

[53] Mantziki K, Vassilopoulos A, Radulian G ea. Inequities in energy-balance related behaviours and family environmental determinants in European children: baseline results of the prospective EPHE evaluation study. Bmc Public Health. 2015;15(1):1–13. doi: 10.1186/s12889-015-2540-5 26630926 PMC4668694

[54] Krist L, Bürger C, Ströbele-Benschop N, Roll S, Lotz F, Rieckmann N, et al. Association of individual and neighbourhood socioeconomic status with physical activity and screen time in seventh-grade boys and girls in Berlin, Germany: a cross-sectional study. BMJ open. 2017;7(12):e017974. doi: 10.1136/bmjopen-2017-017974 29288179 PMC5770905

[55] Yi X, Fu Y, Burns R, Ding M. Weight Status, Physical Fitness, and Health-Related Quality of Life among Chinese Adolescents: A Cross-Sectional Study. International Journal of Environmental Research and Public Health. 2019;16(13):2271. doi: 10.3390/ijerph16132271 31252581 PMC6651867

[56] J I, D C, T Y. Health behaviour in school-aged children (HBSC) study: international report from the 2013/2014 survey. 2016.

[57] Wang X, Liu QM, Ren YJ, Lv J, Li LM. Family influences on physical activity and sedentary behaviours in Chinese junior high school students: a cross-sectional study. BMC Public Health. 2015;15(1):1–9. doi: 10.1186/s12889-015-1593-9 25884212 PMC4376336

[58] Weir LA, Etelson D, Brand DA. Parents’ perceptions of neighborhood safety and children’s physical activity. Preventive Medicine. 2006;43(3):212–7. doi: 10.1016/j.ypmed.2006.03.024 16712912

[59] Hanson MD, Chen E. Socioeconomic status, race, and body mass index: the mediating role of physical activity and sedentary behaviors during adolescence. Journal of pediatric psychology. 2006;32(3):250–9. doi: 10.1093/jpepsy/jsl024 16896193

[60] Stenhammar C, Sarkadi A, Edlund B. The role of parents’ educational background in healthy lifestyle practices and attitudes of their 6-year-old children. Public Health Nutrition. 2007; 10(11): 1305–1313. doi: 10.1017/S1368980007696396 17381933

[61] Chowhan J, Stewart JM. Television and the behaviour of adolescents: Does socio-economic status moderate the link? Social Science & Medicine. 2007;65(7):1324–36. doi: 10.1016/j.socscimed.2007.05.019 17587476

[62] Gordon-Larsen P, Nelson MC, Popkin BM. Longitudinal physical activity and sedentary behavior trends: adolescence to adulthood. American journal of preventive medicine. 2004;27(4):277–83. doi: 10.1016/j.amepre.2004.07.006 15488356

[63] Matarma T, Tammelin T, Kulmala J, Koski P, Hurme S, Lagström H. Factors associated with objectively measured physical activity and sedentary time of 5–6-year-old children in the STEPS Study. Early Child Development & Care. 2016:1–11.

[64] Link BG, Phelan JC. Social Conditions AS Fundamental Causes of Disease. Journal of Health and Social Behavior. 1995;Spec No(extra issue):80–94. 7560851

[65] Chen ST, Liu Y, Tremblay MS, Hong JT, Tang Y, Cao ZB, et al. Meeting 24-h movement guidelines:Prevalence, correlates, and the relationships with overweight and obesity among Chinese children and adolescents. 2021; 10(3): 349–359. doi: 10.1016/j.jshs.2020.07.002 32679341 PMC8167320

